# Zika virus transmission in Angola and the potential for further spread to other African settings

**DOI:** 10.1093/trstmh/try001

**Published:** 2018-01-31

**Authors:** Moritz U G Kraemer, Oliver J Brady, Alexander Watts, Matthew German, Simon I Hay, Kamran Khan, Isaac I Bogoch

**Affiliations:** 1 Department of Zoology, University of Oxford, Oxford, United Kingdom; 2 Harvard Medical School, Boston; 3 Boston Children’s Hospital Informatics Program, Boston, United States; 4 Centre for the Mathematical Modeling of Infectious Diseases, London School of Hygiene and Tropical Medicine, University of London, United Kingdom; 5 Li Ka Shing Knowledge Institute, St. Michael’s Hospital, Toronto, Canada; 6 Institute for Health Metrics and Evaluation, University of Washington, Seattle, WA, USA; 7 Big Data Institute, Li Ka Shing Centre for Health Information and Discovery, Oxford, United Kingdom; 8 Department of Medicine, Division of Infectious Diseases, University of Toronto, Toronto; 9 Divisions of General Internal Medicine and Infectious Diseases, University Health Network, Toronto, Canada

**Keywords:** *Aedes*, Africa, Air travel, Angola, Globalization, Mosquito, ZIKV

## Abstract

**Background:**

Cases of Zika virus were recently detected in Luanda, Angola, a major travel hub in Africa. The risk of Zika virus transmission throughout the continent from Angola is evaluated.

**Methods:**

Travel volumes were assessed using monthly passenger-level flight data from Luanda to all locations throughout Africa. Travel data was superimposed onto seasonal environmental suitability maps that predict the potential for subsequent Zika virus transmission.

**Results and Conclusions:**

Windhoek, Maputo, Durban and Kinshasa have the greatest potential for Zika virus introduction and transmission during the southern hemisphere summer months, and Nairobi during the northern hemisphere summer months.

## Introduction

Zika virus (ZIKV), originally endemic to parts of Africa and Asia, has now spread throughout most of Latin America and the Caribbean, and has infected millions of individuals. Infections with Zika virus are typically mild, although data emerging from the current epidemic confirmed that the infection is associated with significant birth defects and Guillain–Barré syndrome, and that it can be transmitted sexually. ZIKV has spread internationally due to the movement of infected individuals and autochthonous transmission is now occurring in several regions that have suitable conditions, namely the appropriate climate and *Aedes* mosquito vectors. Although the current Pan-American outbreak strain is caused by the Asian genotype, the virus has recently been detected in at least three African settings: Guinea-Bissau (African genotype), Angola (genotype yet to be determined)^1^ and Cape Verde (Asian genotype). The African ZIKV genotype has also been known to be circulating in West Africa at low levels for the last few decades, possibly supported by a sylvatic cycle,^2^ although there are suggestions that the ZIKV stain currently circulating in the Americas has significantly higher infectivity.^3^ The more recent cases in urban areas of Angola are of potential concern. Angola is a major gateway between Africa and South America with significant connectivity via air travel and is a plausible location for ZIKV re-introduction.^4^ Furthermore, Angola, its neighbours, and much of the continent is vulnerable to arboviral infections year-round due to conducive climate conditions, and many countries have sub-optimal surveillance systems in place for disease detection. Should Angola become a focal point for ZIKV introduction, directing limited public health resources to strengthen surveillance and control measures in that country, while also predicting where subsequent regional and continental transmission may occur could help mitigate ZIKV-related complications.

## Methods

We analysed monthly passenger-level flight data from Luanda (where two ZIKV cases were recently detected)^1^ to all locations throughout Africa. We analysed all airport destinations and corresponding volumes of travellers for the period of October 2015 to September 2016 using worldwide ticket sales data from the International Air Transport Association. To estimate the potential for local virus transmission arising from ZIKV introduction, we then combined predictions of local monthly variation in ZIKV transmission suitability,^4^ and show seasonal variation in local transmission risk for the 10 most visited African cities from Luanda, Angola.

## Results

**Figure 1.**
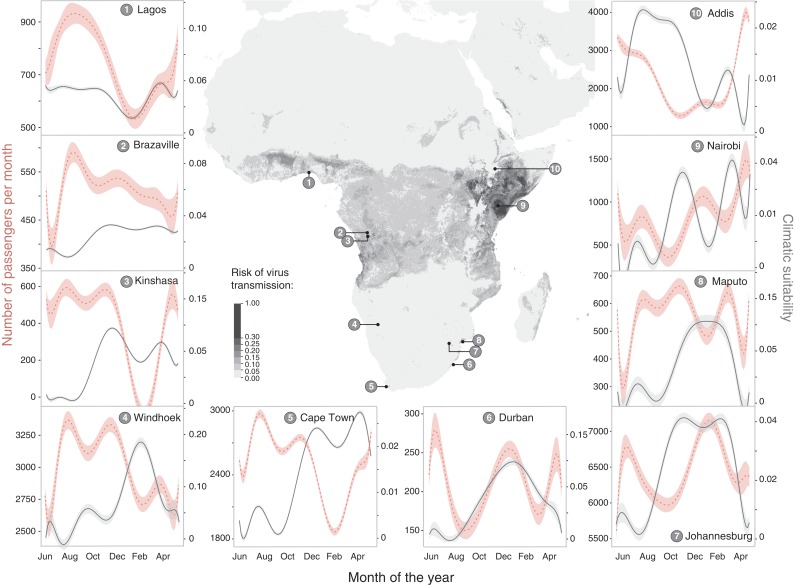
Number of flights per month from Luanda to other African destinations with monthly climatic suitability for Zika virus transmission in passenger-receiving cities. Axes labels show climatic suitability and flight passenger numbers tailored to each location independently to illustrate seasonality and inter-annual variation. Addis Ababa, Cape Town, Johannesburg, Nairobi, and Windhoek have considerably higher numbers of travellers arriving from Angola.

Passengers arriving to African cities conducive to local arbovirus transmission were highest in neighbouring Namibia (Windhoek) followed by Addis Ababa (Ethiopia), which experienced a significant increase in passengers in the months of November to January (Figure 1). Johannesburg and Cape Town (South Africa) both had the highest number of passengers arriving from Luanda, but did not have *Aedes aegypti* suitability for subsequent ZIKV transmission. Areas that have high risk of ZIKV introduction and potential for local transmission are Windhoek, Maputo, Durban and Kinshasa (all during the southern hemisphere summer months, between November and February, inclusive). Transmission potential is expected to shift to the northern hemisphere during their spring and summer months. In this analysis, Nairobi had both the highest potential of ZIKV introduction reflected by the number of arriving passengers coupled with suitable environmental potential for local transmission (Figure 1).

## Discussion

It is currently unclear if the cases of ZIKV detected in Angola are of the African or Asian lineage. Genetic data could help with answering this question and elucidate whether the detection of these cases is indicative of local transmission. If so, there are presumably more unreported cases. This is of concern for pregnant women or women considering pregnancy, given the association between ZIKV infection and foetal complications.^5^ Prior studies evaluating the potential for ZIKV introduction or re-introduction to African and Asia-Pacific settings highlighted Angola as being of increased risk of importation during the months of November to April given the confluence of travel patterns from epidemic locations (from Brazil in particular) and congruent seasonal environmental conditions suitable for ZIKV transmission in the arrival locations of these passengers.^4^ Our results reveal the potential for distant spread of ZIKV from Angola throughout the African continent via air travel, highlighting that Namibia and Mozambique are at highest risk for importation and subsequent transmission. Beyond continental spread via air travel, spread of the virus via land travel is possible, as recently observed with yellow fever cases spreading from Angola to the Democratic Republic of Congo.^6^ Unfortunately, the limited diagnostic, surveillance and public health infrastructure in many at-risk African settings poses several challenges, including providing quality maternal care and mosquito control efforts. Recent advances in rapid and even portable genomic sequencing of arboviruses could improve surveillance, but currently there a dearth of data evaluating the burden of illness in African settings.^7^

It is unclear what the underlying extent of protective immunity to ZIKV exists in African settings. It is also unclear whether the African genotype provides some or any cross-protection against the epidemic Asian strain. These two unknown issues have the potential to significantly influence transmission dynamics of ZIKV in Africa. Regardless of the genotype circulating in Angola, these recently discovered cases have been suggested to be the result of local transmission. This further highlights the potential of arboviruses to spread locally and regionally in African settings, requiring a coordinated public health response.

## Supplementary data

Supplementary data are available at Transactions online (http://trstmh.oxfordjournals.org/).

## Supplementary Material

Supplementary Data

Supplementary Data

Supplementary Data

Supplementary Data

Supplementary Data
